# HIV transmission dynamics and population-wide drug resistance in rural South Africa

**DOI:** 10.21203/rs.3.rs-3640717/v1

**Published:** 2023-11-28

**Authors:** Ravindra Gupta, Steven Kemp, Kimia Kamelian, Diego Cuadros, Ravindra Gupta, Mark Cheng, Elphas Okango, Willem Hanekom, Thumbi Ndung’u, Deenan Pillay, David Bonsall, Emily Wong, Frank Tanser, Mark Siedner

**Affiliations:** University of Cambridge; University of Oxford; University of Cambridge; University of Cincinnati; University College London; cambridge university; AHRI; University of KwaZulu-Natal; UCL; oxford university; Africa Health Research Institute; Stellenbosch University; Harvard Medical School

## Abstract

Despite the scale-up of antiretroviral therapy (ART) in South Africa, HIV-1 incidence remains high. The anticipated use of potent integrase strand transfer inhibitors and long-acting injectables aims to enhance viral suppression at the population level and diminish transmission. Nevertheless, pre-existing drug resistance could impede the efficacy of long-acting injectable ART combinations, such as rilpivirine (an NNRTI) and cabotegravir (an INSTI). Consequently, a thorough understanding of transmission networks and geospatial distributions is vital for tailored interventions, including pre-exposure prophylaxis with long-acting injectables. However, empirical data on background resistance and transmission networks remain limited. In a community-based study in rural KwaZulu-Natal (2018–2019), prior to the widespread use of integrase inhibitor-based first-line ART, we performed HIV testing with reflex HIV-1 RNA viral load quantification on 18,025 participants. From this cohort, 6,096 (33.9%) tested positive for HIV via ELISA, with 1,323 (21.7%) exhibiting detectable viral loads (> 40 copies/mL). Of those with detectable viral loads, 62.1% were ART-naïve, and the majority of the treated were on an efavirenz + cytosine analogue + tenofovir regimen. Deep sequencing analysis, with a variant abundance threshold of 20%, revealed NRTI resistance mutations such as M184V in 2% of ART-naïve and 32% of treated individuals. Tenofovir resistance mutations K65R and K70E were found in 12% and 5% of ART-experienced individuals, respectively, and in less than 1% of ART-naïve individuals. Integrase inhibitor resistance mutations were notably infrequent (< 1%). Prevalence of pre-treatment drug resistance to NNRTIs was 10%, predominantly consisting of the K103N mutation. Among those with viraemic ART, NNRTI resistance was 50%, with rilpivirine-associated mutations observed in 9% of treated and 6% of untreated individuals. Cluster analysis revealed that 20% (205/1,050) of those sequenced were part of a cluster. We identified 171 groups with at least two linked participants; three quarters of clusters had only two individuals, and a quarter had 3–6 individuals. Integrating phylogenetic with geospatial analyses, we revealed a complex transmission network with significant clustering in specific regions, notably peripheral and rural areas. These findings derived from population scale genomic analyses are encouraging in terms of the limited resistance to DTG, but indicate that transitioning to long-acting cabotegravir + rilpivirine for transmission reduction should be accompanied by prior screening for rilpivirine resistance. Whole HIV-1 genome sequencing allowed identification of significant proportions of clusters with multiple individuals, and geospatial analyses suggesting decentralised networks can inform targeting public health interventions to effectively curb HIV-1 transmission.

## Introduction

South Africa remains at the epicentre of the global HIV-1 pandemic, with an estimated 7.5 million people, or 18.3% [15.6–20.5%] of the population, living with HIV-1 as of 2021^1^. Over the last two decades there has been a reduction in the annual incidence of new infections, from 510,000 to approximately 210,000, resulting from an unprecedented public health effort to increase access to antiretroviral therapies^1^. This progress is noteworthy given the scale of the epidemic and highlights the effectiveness of concerted public health efforts. Nonetheless, HIV continues to pose significant morbidity and mortality risks, underscoring the need for ongoing vigilance and intervention^2^.

A principal challenge in the management and control of HIV-1 has been the selection and spread of drug resistance^3^, with a noted global increase in non-nucleoside reverse transcriptase inhibitor (NNRTI) pre-treatment drug resistance (PDR)^4–6^ prompting the World Health Organisation (WHO) to revise its first-line therapy guidelines. This issue is particularly pronounced in sub-Saharan Africa, where NNRTI-based regimens have been compromised by systemic public health failures, especially in regions with suboptimal viral load monitoring^5,7^.

In response to these challenges, there has been a strategic pivot towards the use of integrase strand transfer inhibitors (INSTIs) such as dolutegravir (DTG) in first-line ART regimens^8^. This shift represents a critical evolution in ART strategy, but it also necessitates robust surveillance to monitor the emerging resistance to these newer drugs. Policy now recommends that new patients start on DTG-based ART and those previously treated with viral loads under 1000 copies/mL switch to a TDF/3TC/DTG regimen. Although reports suggest a low prevalence of DTG resistance in ART-naïve individuals^9^, there is a greater risk of INSTI resistance amongst patients viraemic at time of switch to DTG-based ART^10^. Previous resistance to lamivudine or tenofovir may also adversely impact outcomes^11^, with evidence also showing that existing NNRTI resistance is associated with suboptimal responses to INSTI-based regimens^12^.

Understanding the degree and characteristics of population level NNRTI resistance is still of importance given that long acting (LA) Cabotegravir/Rilpivirine treatment is on the horizon^13^, especially given the widespread use of NNRTI in sub–Saharan Africa. Therefore, knowledge around the background prevalence of NNRTI resistance mutations relevant for Rilpivirine in naïve and treated populations remains important.

Another forthcoming LA-regimen combines the capsid maturation inhibitor lenacapavir with the novel NRTI islatravir. Lenacapavir resistance is easily selected in vitro, and the efficacy of islatravir is compromised by the M184V resistance mutation. While there is substantial literature on M184V from high-income countries^14–16^, updated data on the mutation’s prevalence in recent cohorts from high-prevalence settings is critical for islatravir’s potential use^17,18^. Yet, such empirical data on background resistance at the population level remain scarce in high-burden areas like South Africa.

Despite these advancements in ART, the HIV epidemic’s landscape is heterogeneous. High HIV incidence rates persist in specific areas, like the uMkhanyakude district of KwaZulu-Natal (KZN), particularly among adolescent girls and young women^19^. This highlights the necessity for interventions tailored to the local epidemiology. Systemic challenges, including the shortage of community healthcare workers in KZN, have led to failures in care linkage, prompting the need for localised solutions^20,21^. The Vukuzazi programme, initiated in 2018, exemplifies such an approach, targeting HIV, tuberculosis, hypertension, and diabetes prevalence in the community while facilitating direct linkage to care^22^.

This study’s objectives are twofold: to characterise antiretroviral resistance in this largely rural population via whole genome HIV-1 deep sequencing, and to map the potential HIV transmission networks and their geospatial distribution.

## Methods

### Study setting and recruitment

The Vukuzazi study recruited 18,025 participants (adolescents and adults aged ≧ 15) from their homes in the uMkhanyakude district, KwaZulu-Natal, South Africa to healthcare screening for hypertension, diabetes, HIV, and tuberculosis. Full details of the Vukuzazi study methods and results have been previously reported^22,23^. Recruitment occurred between May 25, 2018, and November 28, 2019, during the Vukuzazi cross-sectional survey. Ethical clearances were obtained from the University of KwaZulu-Natal Biomedical Research Ethics Committee, the London School of Hygiene & Tropical Medicine Ethics Committee, and the Partners Institutional Review Boards. All participants provided informed consent for HIV testing and ensuing analysis.

### Date and Blood Sample Collection

Mobile health clinics across the study area facilitated data collection. Research nurses compiled participants’ medical histories, including prior HIV, tuberculosis, hypertension, and diabetes diagnoses. Blood samples from 17,949 participants were collected for HIV testing using the Genscreen Ultra HIV Ag-Ab enzyme immunoassay [Bio-Rad]. The HIV-1 RNA viral load was subsequently measured for immunoassay-positive samples, resulting in 6093 successful tests. Samples with detectable viral load >40 copies/ml (n = 1323) underwent whole-genome sequencing at the University of Oxford.

### Whole genome sequencing and bioinformatics

We employed the veSEQ-HIV^24^ method for whole-genome sequencing on the Illumina MiSeq platform at the University of Oxford, following established protocols. We then used a bioinformatics pipeline, drmSEQ, to identify genotypic resistance, aligning codons with a database of 142 HIV reference sequences. We used the Stanford HIV Drug Resistance Database classification system, with ‘high-level resistance’ as our threshold for resistance. We obtained the participants’ ART status (ART-experienced, n = 583, and ART-naïve, n = 467) from the PANGEA consortium, TIER.net, and the Vukuzazi cohort study. HIV-1 subtyping was done using SNAPPy v1.0. Prediction of co-receptor usage was made using TROPHIX (prediction of HIV-1 tropism). Available at: http://sourceforge.net/projects/trophix/). In addition, we aligned whole genome sequences from the Vukuzazi study with publicly available sequences from the ANRS TasP 12249 study conducted in a neighbouring region in KZN, South Africa in 2012.

### Transmission Cluster Identification and Validation

After inferring a maximum-likelihood phylogeny using IQ-TREE v2.2.2 (1000 ultrafast bootstraps and a GTR + F + R6 model), we utilized ClusterPicker (v1.2.5) to identify potential transmission clusters, yielding 171 clusters. We further refined these results using a backward stepwise logistic regression model based on collection date intervals and patristic distances between sequence pairs, validated via a logit model. This refinement pruned the total number of clusters to 86, of which 75% were linked pairs and the rest consisted of 3–6 linked participants.

### Geospatial Data Visualisation

We executed geospatial visualizations to describe the spatial distribution of several epidemiological parameters. We introduced a geographical random error to uphold participant confidentiality. We evaluated the prevalence of several epidemiological parameters by generating continuous surface maps, utilising a standard Gaussian kernel interpolation method^25^. Maps were created without geographical references, and a grid consisting of 108 hexagonal cells covering the surveillance area was used to aggregate the spatially interpolated estimates. The grid was further employed to illustrate HIV prevalence, treatment failure prevalence, NNRTI mutations, NRTI mutations, and the 163 identified phylogenetic transmission linkages that included information for geolocation. Bivariate maps were generated to identify regions with overlapping epidemiological measures, such as high HIV prevalence coinciding with high rates of treatment failure or significant drug resistance mutations. We geospatially mapped the linkages of viral transmission among these individuals that were estimated by the previous phylogenetic analyses using the grid to aggregate the locations of these links into the centroids of each of the 108 cells of the grid that represented the nodes of the transmission links. We used the software ArcGIS Pro 3.1 (ESRI: ArcGIS Pro.x. Redlands, CA, USA: ESRI. 2020.) to construct the grid and generate the spatial data visualizations included in the study.

## Results

### Study population characteristics

**Figure 1:**
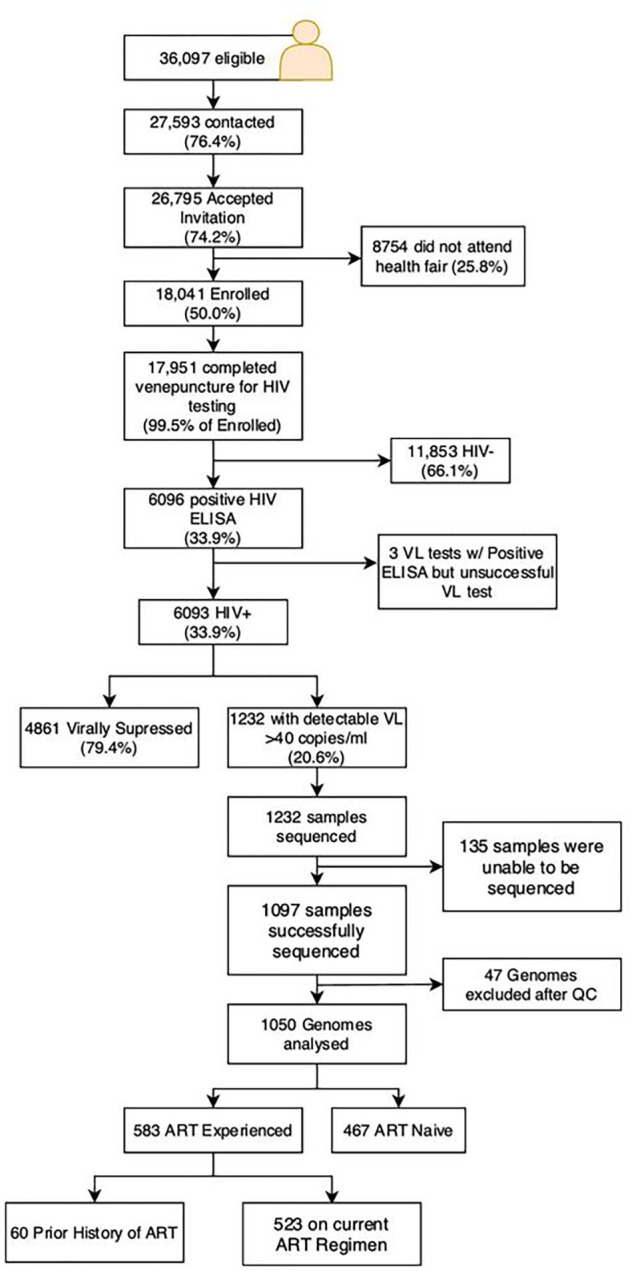
Flow Diagram of Vukuzazi cohort participation. Individuals aged ≥15 years in the Africa Health Research Institute (AHRI) Demographic and Health Surveillance catchment area were eligible for the Vukuzazi study. From 1323 blood samples, 1057 genomes were successfully obtained from the cohort, 36 were excluded due to poor coverage of pol and env genes. Downstream resistance and transmission analysis was therefore conducted with 467 ART-naïve patients and 583 ART-experienced participants.

The Vukuzazi study enrolled 18,025 individuals, with 17,949 (99.6%) completing venepuncture for HIV testing (refer to Fig. 1). Among them, 6,096 (33.9%) tested positive for HIV via ELISA testing. Notably, three participants had a positive HIV ELISA result but no viral load (VL) due to testing issues. The study’s enrolment ratio was 2:1 female to male (Table 1). Approximately two-thirds (62.1%) of those with a positive HIV ELISA were ART naïve at recruitment, based on the study’s metadata and corroborated by electronic health record data. More than 85% of the known treatment regimens comprised EFV/FTC/TDF combination therapy.

Of the 1,232 venous blood samples with a VL > 40 copies/ml, 1,097 resulted in successful whole genome sequences using Illumina’s deep sequencing platform. Post-quality control, 47 genomes were excluded due to low or incomplete genome coverage. This left 467 ART naïve and 583 ART experienced participants for resistance and transmission analysis with high genome coverage (Supplementary Fig. 2). Among the ART-experienced group, 27 individuals were on protease inhibitor-based second-line regimens, and 10 were on dolutegravir-based ART.

### HIV-1 drug resistance

**Figure 2:**
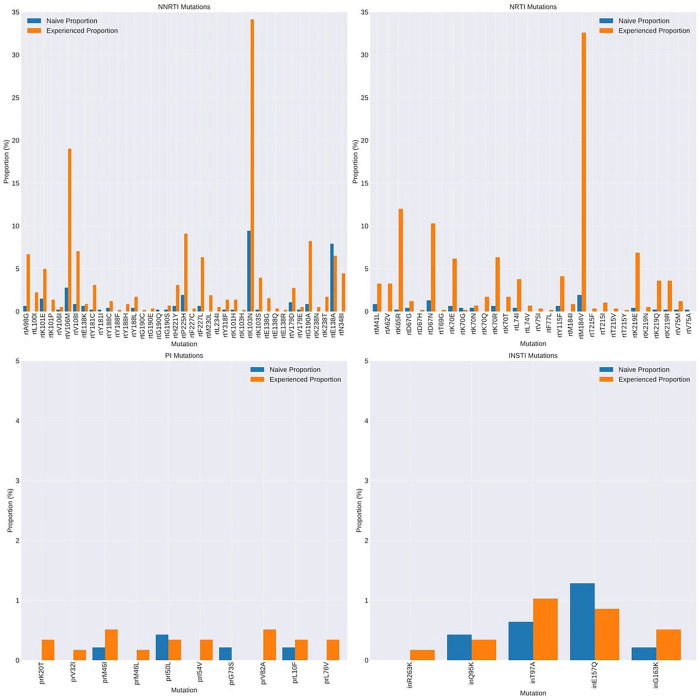
Prevalence of HIV-1 Drug resistance associated mutations at >20% abundance by NGS in treatment naïve and experienced individuals. Data are shown by drug class. NRTI – nucleoside reverse transcriptase inhibitor; NNRTI - non- nucleoside reverse transcriptase inhibitor; INSTI – integrase strand transfer inhibitor; PI – protease inhibitor

At a variant abundance threshold of 5%, common NRTI mutations included M184V, associated with lamivudine and abacavir resistance, observed in 32.6% of treated and 1.9% of naïve individuals (Figs. 2 and Supplementary Figs. 3–4). Tenofovir resistance mutations K65R and K70E were noted in 12.0% and 6.2% of ART-experienced participants, and in < 1% of both ART-experienced and ART-naïve individuals. Thymidine Analogue Mutations (TAMs) including M41L, T215Y, D67N, K70R, T215F, and K219R/Q were all found in < 10% of treated individuals and < 2% of naïve individuals. These findings, demonstrating the presence of TAMs in a largely thymidine analogue-naïve population treated with first-line tenofovir-containing regimens, align well with studies across Sub-Saharan Africa evidencing thymidine resistance in similar regimens^26^.

**Figure 3:**
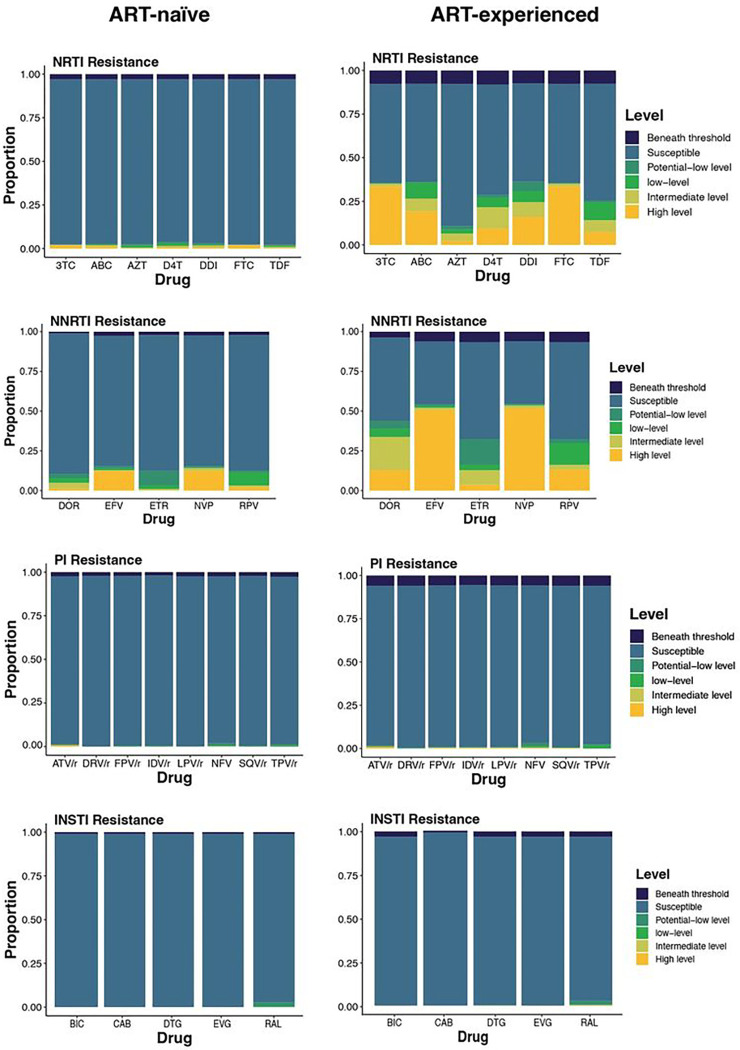
Susceptibility to ART among ART-naïve (left) and ART-experienced (right) participants. Beneath threshold: less than half of the sites had sufficient data at the stipulated threshold to determine resistance. Susceptible: all relevant sites had reads at the stipulated threshold and no mutations were detected. Data for four drug classes are shown. NNRTI - non- nucleoside reverse transcriptase inhibitor; INSTI – integrase strand transfer inhibitor; PI – protease inhibitor

Among the detected NNRTI mutations, the prevalence in ART-experienced versus ART-naïve participants was as follows: K101E was found in 5.0% of ART-experienced and 1.5% of ART-naïve participants; K103N in 34.1% of ART-experienced and 9.4% of ART-naïve; V106M in 32.6% of ART-experienced and 2.8% of ART-naïve; and G190A in 8.2% of ART-experienced compared to 0.9% of ART-naïve participants. Importantly, the E138A mutation, known for conferring cross-resistance to rilpivirine - a second-generation NNRTI used in long-acting injectable ART alongside cabotegravir - (Figs. 3, Supplementary Figs. 3–4), was observed in 6.5% of ART-experienced participants and 7.9% of ART-naïve participants (Fig. 2).

Resistance-associated mutations for integrase strand transfer inhibitors (INSTIs) and protease inhibitors (PIs) were rare in both ART-treated and naïve groups (Figs. 2 and 3). Both were found in fewer than 10 participants in the ART-treated and naïve groups. The most common INSTI mutations were T97A and E157Q (Fig. 2, both at less than 1.5%). Both mutations are previously reported as polymorphic, although T97A is associated with high-level resistance to DTG when in the presence of major INSTI mutations such as G140S (https://hivdb.stanford.edu).

Analysis of low frequency NRTI variants between 2 and 20% (Supplementary Fig. 3) showed that only K65R, K70N, and K219R were detected in treated individuals. M184V was only observed at high variant abundance in both treated and naïve individuals, with the caveat that M184V occurs rarely in naïve individuals. Integrase polymorphism E157Q was observed only at high variant abundance both in treated and naïve individuals (Supplementary Fig. 5), consistent with low fitness cost of this mutation.

Finally, coreceptor usage was analysed using deep sequence data in the V3 loop region. The majority of viruses (93.8%) exhibited predicted CXCR5 co-receptor usage.

### HIV-1 Transmission Clusters

**Figure 4:**
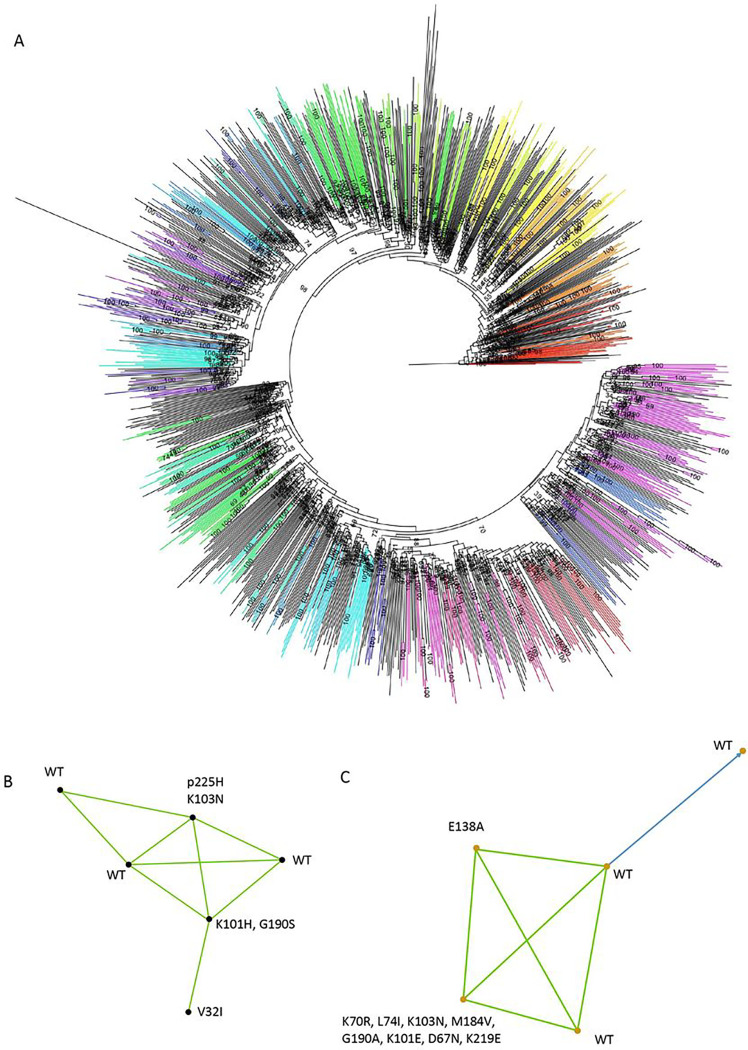
Phylogenetic analysis of HIV-1 sequences and identification of transmission clusters in the Vukuzazi cross sectional study A. Maximum likelihood phylogenetic tree of HIV-1 sequences that passed QC. Bootstrap support indicated at nodes. Clusters as defined by Cluster picker using genetic distance threshold of 4.5 and statistical support by bootstrapping of >98% are coloured. B. Linkage and resistance data on largest cluster with 6 participants and C. Linkage and resistance data on second largest cluster with 5 participants. Blue lines indicate >95% confidence and green indicate >80% confidence.

We identified a total of 171 clusters in total with at least two linked participants (Fig. 4). 205/1050 (19.5%) of participants with sequence data were part of a cluster. 75% of clusters contained two individuals and the remaining 25% of clusters comprised 3–6 individuals (Fig. 4B–C). In the largest cluster of 6 participants, three had evidence of drug resistance (Fig. 4B) and three were wild type. In the second largest cluster with five participants, three were wild type, one had only the NNRTI mutation E138A and the fifth had multiple NNRTI and NRTI mutations (Fig. 4C). There was strong evidence for directionality between two of the individuals in this cluster (Fig. 4C, blue line with arrow).

We next examined the Vukuzazi sequences in the context of older whole genome sequences from 2011/12 from a neighbouring geographical area in KZN that were collected as part of the ANRS12249 Treatment as Prevention Trial. We hypothesised that there would be linked infections between the two studies due to proximity of the study areas, and indeed TasP and Vukuzazi sequences were dispersed and intermingled throughout a maximum likelihood phylogenetic tree (Supplementary Fig. 6A). Cluster analysis revealed presence of linked infections involving TasP and Vukuzazi participants (Supplementary Fig. 6B,C and Supplementary Fig. 7).

### Geospatial visualisations

The geospatial structure of epidemiological measures such as HIV viremia on treatment and drug resistance are graphically presented in Fig. 4. A varying level of HIV prevalence across the region with predominantly high HIV prevalence (> 35%) is observed in the bottom right (southeast) portion of the sampled area (dark blue regions in Fig. 4A), whereas the prevalence of treatment failure does not exhibit a clear spatial pattern (Fig. 4B). Notably, the bivariate map delineating HIV prevalence and treatment failure identifies areas bearing a high burden of both measures (dark green in Fig. 4C), primarily concentrated in regions previously marked with high HIV prevalence. The geographical distribution of NNRTI (Fig. 4D) and NRTI (Fig. 4E) resistance prevalence reveals distinct patterns: NNRTI resistance is found primarily in the northern and southern surveillance regions, while NRTI resistance is more common in the northern region. The bivariate map presenting both mutations’ prevalence identifies overlapping areas in the southern, central, and northern parts of the survey area (dark blue in Fig. 4F), as well as regions with high-NRTI-low-NNRTI prevalence (dark red) and low-NRTI-high-NNRTI prevalence (bright blue).

**Figure 5:**
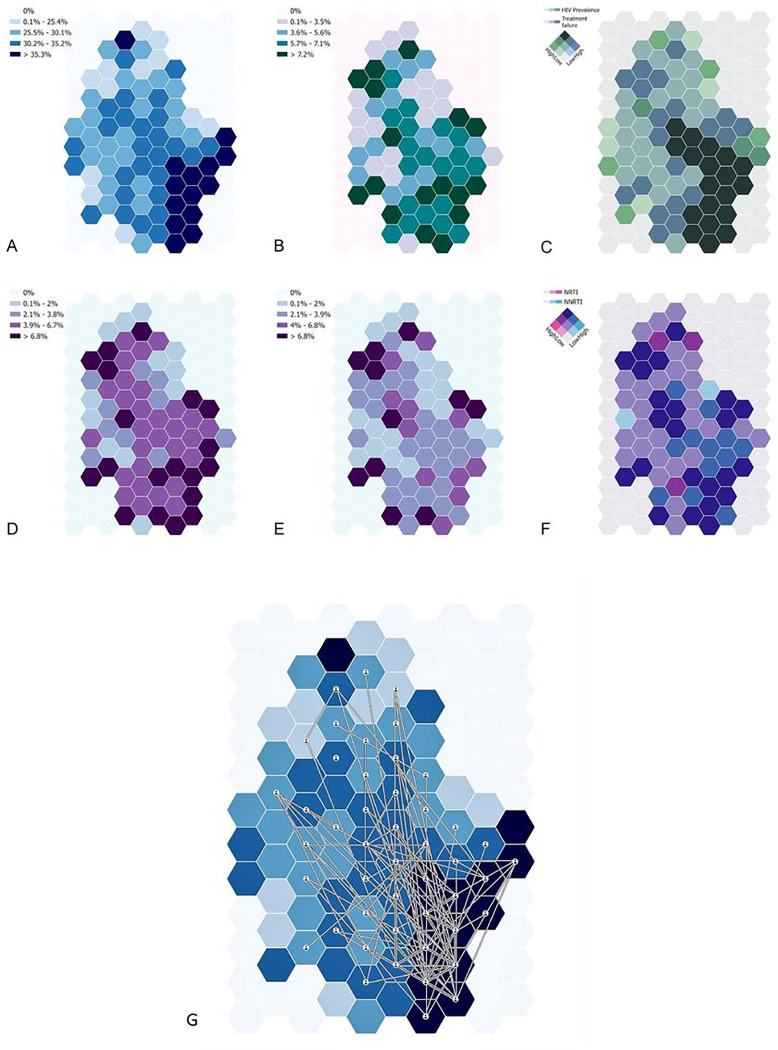
Geospatial analysis of NRTI and NNRTI resistance in the uMkhanyakude district. A) HIV prevalence; B) prevalence of treatment failure among HIV positive individuals; C) bivariate map among HIV prevalence and treatment failure; D) NRTI prevalence among HIV positive individuals; E) NNRTI prevalence among HIV positive individuals; F) bivariate map among NNRT and NNRTI. G) High confidence phylogenetically-linked participants plotted on the background of HIV prevalence.

Phylogenetic linkages appear predominantly in the central and southern parts of the survey area showing high connectivity within these regions, largely coinciding with regions of high HIV prevalence. Highly connected nodes, ranging from 21 to 69 connections, are situated in the southern part of the survey area where HIV prevalence is higher. Nodes with fewer connections (4–9) are mainly found in the central region of the survey area where HIV prevalence is lower, as might be expected (Fig. 5).

### Role of the Funding Source

The funding sources for this study had no role in the study design, data collection, data analysis, data interpretation, or writing of the report.

## Discussion

ART treatment guidelines currently recommended the fixed dose combination (FDC) of tenofovir, lamivudine, and dolutegravir (TLD) as the first-line regimen for all eligible adults, adolescents, and children aged > 10^27,28^. While the WHO has endorsed DTG-based ART as the preferred first-line regimen, understanding the patterns of NRTI and NNRTI antiretroviral resistance in both ART-naïve and ART-experienced patients remains crucial. This is particularly pertinent in the context of increasing evidence that NNRTI Pre-therapy Drug Resistance (PDR) is associated with reduced efficacy and long-term failure of INSTI-containing first-line regimens.^12^.

Currently, long-acting injectables are centred around cabotegravir and rilpivirine (CAB/RPV). Reports suggest a 7.9% prevalence of RPV resistance mutations in ART-naïve populations, with a slightly higher 9.3% in treated population^29^. RPV mutations have also been associated with virologic failure of LA CAB/RPV^30^. Similarly, we observed a high prevalence of intermediate or high level RPV resistance at around 10% in treated participants and 5% in untreated. These findings suggest that ART strategies involving switch to long acting cabotegravir (INSTI) + rilpivirine (NNRTI) may need to include prior screening for rilpivirine resistance.

Regarding the future use of islatravir, the prevalence of M184V was significant in those viremic on ART, and present at lower levels in untreated individuals. M184V has been associated with reduced replication efficiency making it uncommon as a transmitted resistance mutation^16,26,31^. However, compensatory mutations such as L74I in the reverse transcriptase (RT) gene can restore virus fitness in the presence of M184V^18^, therefore, the persistence of M184V poses a risk to the future use of lenacapavir and islatravir long-acting ART regimens.

K65R, a mutation that confers intermediate to high-level tenofovir resistance, was observed in 15% of ART-treated viraemic individuals. This was the only mutation noted as a minority population in ART-naïve individuals, reflecting findings from an earlier study in South Africa^32^. This trend is likely due to the known propensity of subtype C to acquire this mutation spontaneously^33–36^, attributable to differential pausing of RT at positions 64–66^37^.

Before the scaling up of DTG, significant NRTI and NNRTI resistance were identified, but resistance to protease inhibitors (PI) and INSTI were minimal, as expected (Table 2 shows participants with PI or INSTI resistance). However, low-level appearances of INSTI mutations within quasi-species were detected, including R263K - a known DTG-resistance-associated mutation in both B and non-B subtype viruses - among other secondary Drug Resistance Mutations (DRMs)^38,39^. The T97A mutation, a secondary drug resistance mutations (DRMs)^40^, was also observed. This can greatly enhance resistance in the presence of other mutations. Ongoing surveillance for INSTI resistance in the DTG treatment era is critical, given the highly promising results from cabotegravir PreP studies^41,42^.

The application of next-generation sequencing for full-length HIV-1 genomes allowed us to conduct clustering analysis, revealing more than 170 clusters, predominantly involving sequences without DRMs. This implies that transmission occurs before the initiation of ART. Geospatial analyses showed that most clustering occurred in areas with highest prevalence of HIV infection, and also indicated that most clustering happened in areas with the highest HIV infection prevalence. Drug resistance to NNRTI - but not NRTI -coincided with HIV-1 prevalence, possibly due to the significant presence of transmitted NNRTI resistance in this population. Conversely, both NRTI and NNRTI resistance were geospatially associated with treatment failure, reflecting the high probability of detecting NRTI and NNRTI resistance following treatment failure.^7^.

Building on previous study methodologies^43^, our geospatial data visualisation provided a nuanced view of HIV prevalence, treatment failure, and drug resistance distribution. Despite the scale-up of ART, high HIV infection burdens and considerable levels of treatment failure were evident, signalling ongoing transmission and potential drug resistance emergence. This information could be vital for crafting effective public health strategies by pinpointing resource allocation for testing, treatment, and prevention efforts.

High-resolution mapping of HIV prevalence, treatment failure, and drug-specific resistance offers insights into the potential for decentralised sampling in surveillance programmes. Regions marked by high HIV prevalence and ART failure require a robust response, including expanded testing initiatives. Mobile testing units and community health workers could be strategically placed in these high-need areas, potentially increasing diagnosis rates and linking individuals to treatment services more effectively. The maps can also guide adherence support programmes to regions where treatment failure is prevalent. By integrating different strategies, such as digital adherence tools, peer support groups, and community-based interventions, healthcare systems can aim to enhance patient outcomes and mitigate the burden of treatment failure^44^. This geospatial data can empower healthcare providers to optimise treatment by selecting ART combinations less susceptible to resistance.

The spatial dynamics of HIV transmission provide insight into the epidemic’s spread. Identifying highly connected zones allows for targeted prevention efforts, such as increased condom distribution, education campaigns, and PrEP for high-risk individuals^45^. Understanding these interlinkages enables interventions to be more precisely aimed at community locations central to disease spread^43,46^. The high geospatial resolution transmission linkages identified, coupled with the distribution of drug-class mutations, can inform contemporary local prescribing decisions and policy, particularly as we progress towards long-acting injectables. Thus, we advocate for ART programmes to be coupled with viral load and drug resistance monitoring using NGS in order to enable clustering analyses to be scaled up^47^.

The observed geospatial patterns of HIV are influenced by various factors, including mobility and migratory patterns^48^. Although study participants were effectively linked to healthcare services, the potential for HIV transmission during travel or migration persists, leading to decentralised transmission patterns. Social and sexual network dynamics are equally crucial, as HIV transmission is largely influenced by these structures, often extending beyond geographical limits. The distinct spatial distribution of NNRTI and NRTI resistance observed in the study is likely influenced by several factors, such as the historical deployment and use duration of these antiretroviral drugs within the community^49–51^.

The Vukuzazi study’s unique population-based approach, as opposed to clinic-based or smaller cohort studies, provided a comprehensive and less biased snapshot of the current HIV resistance landscape. Sequencing a large number of community samples offers a detailed picture of the HIV resistance landscape, critical for designing interventions and tracking transmission networks, offering a detailed perspective that is often lost in smaller, more selective sampling methods. This large-scale analysis not only enables more precise detection of prevalent resistance mutations but also facilitates more accurate inference and spatial geolocation of transmission networks within the community. Such granularity in data is rare and instrumental in shaping effective public health strategies and interventions.

Limitations of this study include its cross-sectional nature and capturing resistance at a single time point rather than over an infection course^52^. Ideally serial surveys would be undertaken to provide information on dynamics, particularly in the DTG era. Furthermore, the data on treatment history required collation from more than one source and in some cases were incomplete. In addition, the small sample sizes in some locations make it difficult to draw robust conclusions regarding the geographic patterns of resistance across all parts of the study area.

In conclusion, our study delivers novel insights into the patterns of HIV drug resistance and linkages within a rural area of very high HIV prevalence. HIV-1 whole genome deep sequencing has facilitated not only clustering analyses, but also a more intricate understanding of the HIV-1 drug resistance landscape at population scale.

## Figures and Tables

**Table 1.** Population characteristics, ART status and regimens. Data from 1202 participants with associated metadata for whom blood samples were collected and sent to University of Oxford for sequencing. Of those, 1050 were successfully analysed here.

	All (n=1050)	ART-Naïve (n=467)	Art-Experienced (n=583)
**Age Group (years)**			
15–24	218 (20.8%)	116 (24.8%)	102 (17.5%)
25–34	378 (36.0%)	189 (40.5%)	189 (32.4%)
35–44	261 (24.9%)	99 (21.2%)	162 (27.8%)
45–54	147 (14.0%)	101 (21.6%)	46 (7.9%)
>55	74 (7.0%)	45 (9.6%)	29 (5.0%)
**Sex**			
Female	693 (66.0%)	298 (63.8%)	395 (67.8%)
Male	357 (34.0%)	169 (36.2%)	188 (32.8%)
**Currently on ART?**			
Yes		-	603*
No		-	34
Unknown		-	565
**ART Regimen**			
**NRTI + NNRTI**			348 (89.5%)
TDF, FTC, EFV		-	329
TDF, EFV, 3TC		-	5
AZT, EFV, 3TC		-	5
ABC, EFV, 3TC		-	1
d4T, EFV, 3TC		-	1
TDF, FTC, EFZ		-	1
TDF, NVP, 3TC		-	3
AZT, NVP 3TC		-	2
TDF, FTC, NVP		-	1
**NRTI + PI**		-	27 (6.9%)
TDF, 3TC, LPV		-	14
AZT, 3TC, LPV		-	9
ABC, 3TC, LPV		-	2
TDF, FTC, LPV		-	1
TDF, ATV, LPV		-	1
**NRTI + INSTI**		-	10 (2.6%)
TDF, 3TC, DTG		-	10

* Participant is currently taking ART, but regimens were unknwon in 274 participants.

Abbreviations: EFV, Efavirenz; FTC, Emtricitabine; TDF, Tenofovir; 3TC, lamivudine; LPV/r, Lopinavir/Ritonavir; DTG, Dolutegravir; d4T, stavudine.

**Table 2.** Multi-class drug resistance, and cases of PI and INSTI resistance amongst all participants. Three individuals exhibited three-class resistance. Seven showed PI resistance only. None of these participants existed in a linkage cluster.

ID	ART Class				No. of Classes	VL	ART Status
	NRTI	NNRTI	PI	INSTI			
1	-	-	-	Yes	1	4076	ART Experienced
2	-	-	Yes	-	1	882	ART Experienced
3	-	-	Yes	-	1	21056	ART Experienced
4	-	-	Yes	-	1	155684	ART Experienced
5	-	Yes	Yes	-	2	55526	ART Experienced
6	-	Yes	Yes	-	2	12276	ART Experienced
7	-	Yes	Yes	-	2	2939	ART Experienced
8	Yes	Yes	Yes	-	3	276	ART Experienced
9	Yes	Yes	Yes	-	3	73231	ART Experienced
10	Yes	Yes	Yes	-	3	37752	ART Experienced
11	-	-	Yes	-	1	3360	ART Naïve
12	-	-	Yes	-	1	3817	ART Naïve
13	-	-	Yes	-	1	4531	ART Naïve
14	-	-	Yes	-	1	23279	ART Naïve
15	-	Yes	Yes	-	2	11617	ART Naïve
